# Mathematical modelling of macrophage and natural killer cell immune response during early stages of peritoneal endometriosis lesion onset

**DOI:** 10.1098/rsif.2025.0076

**Published:** 2025-08-13

**Authors:** Claire M. Miller, Domenic P. J. Germano, Alicia M. Chenoweth, Sarah Holdsworth-Carson

**Affiliations:** ^1^ Auckland Bioengineering Institute, The University of Auckland, Auckland, New Zealand; ^2^ School of Mathematics and Statistics, The University of Sydney, Sydney, New South Wales, Australia; ^3^ Breast Cancer Now Research Unit, School of Cancer and Pharmaceutical Sciences, King's College London, London, England, UK; ^4^ St John’s Institute of Dermatology, School of Basic and Medical Biosciences, King's College London, London, England, UK; ^5^ Julia Argyrou Endometriosis Centre, Epworth HealthCare, Richmond, Victoria, Australia; ^6^ Department of Obstetrics, Gynaecology and Newborn Health, The University of Melbourne, Melbourne, Victoria, Australia; ^7^ Department of Obstetrics and Gynaecology, Monash University, Melbourne, Victoria, Australia

**Keywords:** endometriosis, mathematical biology, immune system, macrophages, natural killer cells, computational biology

## Abstract

The immune system is hypothesized to contribute to the onset of endometriosis lesions. However, the precise mechanisms underlying its role are not yet known. We introduce a novel compartmental model that describes the interactions between innate immune cells, specifically macrophages and natural killer cells, and endometrial cells, occurring within the peritoneal fluid during the early stages of (superficial peritoneal) endometriosis lesion onset. Our study focuses on retrograde influx, immune detection and immune clearance. Results show an increased influx of endometrial cells into peritoneal fluid correlates with heightened pro-inflammatory macrophage activation, but does not lead to an increase in disease. We compare the system’s response to changes in immune cytotoxicity and ability to detect ectopic endometrial cells. We predict that reduced cytotoxicity is a key driver of disease. These findings align with the increased immune activation observed clinically. Finally, we predict that an individual can transition to a diseased state following a reduction in immune system cytotoxicity and/or reduced ability to detect ectopic cells. Due to hysteresis, a significant improvement is then required to restore an individual to the disease-free state. This work provides a valuable framework to explore hypotheses of endometriosis lesion onset and assist in understanding of the disease.

## 1. Background

Endometriosis is a chronic gynaecological condition that affects approximately one in nine women [1]. The disease is characterized by the presence of endometrial-like tissue (tissue similar to the lining of the uterus) growing in lesions outside of the uterus. Endometriosis results in a variety of symptoms, including chronic pain and fertility issues [2,3].

Despite its high prevalence, the mechanisms behind the onset of endometriosis remain an open question [1,2]. One hypothesis is Sampson’s theory of retrograde menstruation [2–4]. Under this hypothesis, some menstrual debris, which includes endometrial stromal and epithelial cells as well as immune cells, is cleared from the uterus through the fallopian tubes, rather than through the cervix, during menstruation. After exiting the fallopian tubes, endometrial cells and other menstrual debris collect in the peritoneal fluid (4−16 ml [5,6]) within the peritoneal cavity. Endometrial cells are carried by this fluid and some of these cells may attach to internal tissues, invade the tissue and develop into lesions.

However, studies have found the prevalence of people with retrograde menstruation is greater than the prevalence of endometriosis [7], indicating that the retrograde menstruation theory alone is insufficient to explain disease onset. Supporting this, a recent review has questioned whether previous evidence is sufficient to confirm differences in frequency and volume of retrograde menstruation in patients with endometriosis [8]. This leads to two key hypotheses: (A) in patients with endometriosis, a normally functioning immune system is overcome by an increased concentration of endometrial cells from menstrual debris, or (B) there exists an additional dysfunction of the immune system in those that experience disease. These hypotheses are still open questions for endometriosis pathophysiology [2].

In support of Hypothesis A, studies have shown a decreased proportion of cells undergoing apoptosis within the eutopic endometrium of patients with endometriosis [9], indicating an increased number of viable endometrial cells with the potential to form lesions enter the peritoneal fluid. Endometriosis has also been associated with shorter menstrual cycle length, increased menstrual flow duration and heavy menstrual flow volume [10–12], which all indicate increased influx into the peritoneal cavity. However, other studies have shown no significant difference in clonogenicity or concentrations of endometrial mesenchymal stromal and epithelial stem cells in both peritoneal fluid and menstrual blood between patients with endometriosis and controls, although much higher variation was observed in the patients with endometriosis [13].

Ectopic endometrial cells in the peritoneal cavity should be cleared by the immune system, preventing lesion formation. Consequently, a dysfunction in the ability of the immune system to detect or clear the cells could be involved in disease onset (Hypothesis B) [14]. Several studies have shown differences in immune cell profiles in the peritoneal fluid of patients with endometriosis compared with controls [3,15–18]. Due to the complexity of the immune system, it is difficult from observation alone to identify which differences are causes of immune dysfunction, a consequence of immune dysfunction or simply a consequence of the presence of the endometrial cells. Mathematical modelling enables us to understand the relationship between hypothesized immune dysfunctions and emergent immune system profiles, for example in viral infections [19] and cancer growth [20].

The early immune response is dominated by the innate immune system. Macrophages and natural killer (NK) cells are two innate immune cell types commonly implicated in the endometriosis literature. Macrophages contribute to the inflammatory environment of the peritoneal fluid [21,22] and are involved in the clearance of endometrial cells through phagocytosis and detection of apoptotic markers [23,24]. NK cells help clear endometrial cells through their strong cytotoxic activities [2,3,25].

Macrophages in the peritoneal cavity originate from endometrium, circulating monocytes and tissue-resident macrophages (both monocyte-derived and embryonic-derived). Different macrophage phenotypes have been hypothesized to either contribute to lesion growth (endometrial), or protect against lesion establishment (monocyte-derived tissue-resident) [26]. Traditionally, macrophage activation states have been categorized as *pro-inflammatory* (also known as M1-type or classically activated macrophages) or *pro-repair* (also known as M2-type or alternatively activated macrophages). This binary classification is, for this study, a useful simplification of a macrophage activation spectrum [27,28]. We define pro-inflammatory macrophages as macrophages that secrete inflammatory cytokines and likely contribute to maintaining a level of chronic inflammation that is associated with endometriosis [2,22]. We define pro-repair macrophages as macrophages that are anti-inflammatory and are known to promote lesion growth, through upregulation of angiogenic factors and endometrial cell proliferation [2,29]. For convenience, we refer to pro-inflammatory macrophages as M1-type and pro-repair macrophages as M2-type in this article.

Studies exploring macrophage activation in the peritoneal fluid of patients with endometriosis consistently find an increased proportion of macrophages expressing markers of activation [15–18]. These studies have shown a significant increase in the proportion of cells expressing M2-type markers [16–18]. In eutopic endometrium, increased M2-type activation has been observed to be associated with late-stage disease [30]. Ectopic endometrial stromal cells in co-culture have also been shown to induce macrophage secretion of cytokines associated with the promotion of M2-type activation [22,31,32]. Increases in macrophages expressing M1-type markers in peritoneal fluid have also been observed, but to a smaller (or non-significant) degree compared to M2-type [16–18]. However, observations of increased pro-inflammatory cytokine expression in peritoneal fluid [3,18,21] and increased secretion of IL-1 [3,21] and cytotoxicity of peritoneal macrophages (from peritoneal fluid) in early-stage disease [33] are all associated with an M1-type activation. Cytotoxicity of peritoneal macrophages (from peritoneal fluid) has also been shown to decrease in late-stage (stage III/IV) disease [33]. Together, this suggests M1-type activation may increase in early but not late-stage disease, reflecting an evolving disease environment that could explain the high variability in M1-type activation across patients. Observations of decreased peritoneal macrophage cytotoxicity against both eutopic and ectopic endometrial cells compared with peripheral macrophages in patients with endometriosis [34] could also indicate immune function changes as a response to persistent exposure to an altered immune environment, rather than a pre-existing dysfunction. A potential contributor to the inflammatory peritoneal environment of patients with endometriosis is the increased M1-type activation in eutopic endometrium which has been observed in patients with endometriosis [35]. Endometrial stromal fibroblasts from eutopic endometrium of patients with endometriosis also display pro-inflammatory profiles and progesterone resistance [36]. Additionally, a correlation between activated fibroblasts and macrophage populations has been observed in lesion microenvironments [37]. These observations point to a complex relationship between stromal fibroblasts and immune cells in both cycling endometrium and lesion growth.

Macrophages mediate the clearance of cells through the detection of apoptotic markers and phagocytosis [38], which have been shown to be dysregulated in endometriosis lesion cells [39–41]. Several studies have established that ectopic endometrial tissue does not display the same cyclic changes in apoptotic markers as eutopic tissue [42,43], has a decreased level of apoptosis compared with eutopic endometrium in the same patient [44], and increased anti-apoptotic and decreased pro-apoptotic marker expression compared with eutopic endometrium [42,43,45]. Decreased oestrogen receptor expression is associated with upregulation of anti-apoptotic markers [43]. In patients with endometriosis, decreased [46] and out-of-phase [47] variation in oestrogen receptors on ectopic cells have been observed over the menstrual cycle. In eutopic endometrium, a reduction in spontaneous apoptosis has also been observed [45]. These observations indicate a reduction in the expression of apoptotic markers on these cells, resulting in a decrease in the detection of these cells by the immune system (Hypothesis B1).

NK cells are categorized into two types: CD56${,}^{\text{bright}}$ (also known as CD56${,}^{\text{bright}}$CD16${,}^{-}$) and CD56${,}^{\text{dim}}$ (also known as CD56${,}^{\text{dim}}$CD16${,}^{+}$). CD56${,}^{\text{bright}}$ cells have high cytokine production while CD56${,}^{\text{dim}}$ cells have higher cytotoxicity [14]. In the endometrium, the majority of NK cells are CD56${,}^{\text{bright}}$ [2]. However, the NK cell profiles in peritoneal fluid during normal function are not fully described. There is mixed evidence on the activation of NK cells in peritoneal fluid of patients with endometriosis. After activation by monocytes, NK cells release IFN-γ and TNF-α, the latter of which is consistently raised in patients with endometriosis [14]. However, the activity of NK cells has been shown to be decreased in patients with endometriosis compared with controls [25]. Peritoneal fluid from patients with endometriosis has been shown to reduce cytotoxicity in NK cells *in vitro* [25,48]. Some studies show IFN-γ induces apoptosis in eutopic but not ectopic endometrial cells [14]. This leads to the hypothesis of reduced clearance of the ectopic endometrial cells by NK cells (Hypothesis B2).

In this article, we describe a novel compartmental mathematical model of the immune response to endometrial cells in peritoneal fluid. Endometriosis lesions are defined by depth of infiltration (superficial or deep) and location (peritoneal or ovarian) [47]. Our model focuses on the early stages of superficial peritoneal endometriosis lesion onset and immune response, and consequently, we only consider innate immune cells. We model the effects of NK cells and macrophages, as these are the cells most commonly hypothesized to be involved in endometriosis onset, as detailed above. We focus on the two different immune cell functions and their potential role in disease, as opposed to the roles of different endometrial cell types. Using this model, we investigate two questions around endometriosis onset:

(1) How does the peritoneal fluid immune cell profile change when the amount of retrograde influx is varied, and can increased influx explain observed immune cell profiles in patients with endometriosis and disease onset?
(2) Which immune system disorder: reduced detection or reduced clearance of endometrial cells by the immune system, is most associated with disease and is consistent with the observed immune cell profiles?

## 2. Methods

In this section, we describe the model assumptions governing the interaction between the macrophages, NK cells and endometrial cells, and define three emergent attachment states which dictate the disease-free and diseased states.

### 2.1. Model

We model the interaction between macrophages (M), natural killer cells (K) and endometrial (stromal and epithelial) cells (E) in peritoneal fluid. Each cell type has the following activation states:

— Macrophages are **resting** ($M_{0}$), pro-inflammatory/**M1-type** ($M_{1}$) or pro-repair/**M2-type** ($M_{2}$).
— NK cells are **resting** ($K_{0}$) or **activated** ($K_{A}$).
— Endometrial cells are **eutopic** ($E_{0}$), **in peritoneal fluid** ($E_{F}$) or **attached** ($E_{A}$).

**Figure 1.**
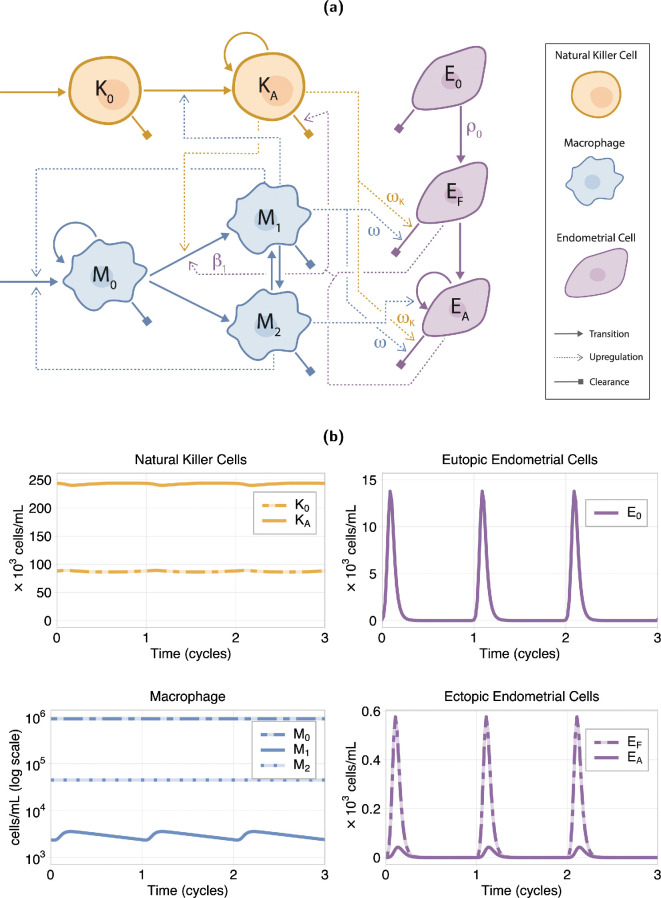
(a) Model diagram with resting ($K_{0}$) and activated ($K_{A}$) NK cells (orange); resting ($M_{0}$), M1-type ($M_{1}$) and M2-type ($M_{2}$) macrophages (blue); and eutopic ($E_{0}$), in fluid ($E_{F}$) and attached ($E_{A}$) endometrial cells (purple). Arrowed solid lines represent cell influx, transition and proliferation; square arrows represent the cell clearance; and dashed lines represent upregulation of a process. The rate parameters of interest for this study are indicated, where $\omega_{K}=\gamma \omega$ and $\omega_{M}=(1-\gamma )\omega$. (b) Endometrial and immune cell dynamics over three menstrual cycles (on average 28 days) using the default parameter values (see electronic supplementary material, S1). The peak in the eutopic endometrial cells, $E_{0}$ (top right of (b)), is the menstrual phase of the cycle and these peaks are reflected in the ectopic endometrial cells, $E_{F}$ and $E_{A}$ (bottom right of (b)). Similar oscillations can also be seen in the immune system cells (left column of (b)). We see all endometrial cells are cleared before the end of each cycle.

We consider attached endometrial cells to be early lesion cells. A diagram of the system model is given in figure 1a and explanations of each of the cell state transitions and cell interactions are given below.

#### 2.1.1. Macrophages

We denote macrophage as resting, $M_{0}$, which includes both monocytes from circulation and tissue-resident macrophage. These can be activated to either a pro-inflammatory M1-type, $M_{1}$ or pro-repair M2-type, $M_{2}$. This binary classification of activated macrophages reflects the average behaviours of a macrophage population activated across a pro-inflammatory to pro-repair spectrum.

*Macrophage turnover*: Macrophage population growth occurs due to: (i) recruitment from the bloodstream (circulating monocytes); or (ii) resting macrophage proliferation (tissue-resident macrophage). We incorporate these two mechanisms in the model as a single growth term. The growth process is upregulated by the presence of activated macrophages ($M_{1}$ and $M_{2}$), and is independent of the activation state. Macrophage cell loss occurs through natural cell turnover from all states.

*Activation of macrophages*: Activated macrophages can be in an $M_{1}$ or an $M_{2}$ state. We assume this occurs through both activation from a resting state and repolarization between the two activated states. We also assume that the cells activate to the pro-repair $M_{2}$ state at a constant rate in early-stage disease, for tissue repair and maintenance, and activate to the inflammatory $M_{1}$ state when they detect the presence of the endometrial cells [30]. $M_{1}$ activation is upregulated by activated NK cells, through the cytokine IFN-γ [14,22], as detailed below.

*Activated macrophage actions*: M1-type macrophages produce pro-inflammatory cytokines, such as interleukins IL-12 and IL-1 [27]. These are known to activate and stimulate production of NK cytokines. We therefore assume $M_{1}$ cells upregulate activation of NK cells. We model M1-type cells as anti-lesion cells that assist in clearance of ectopic endometrial cells, and M2-type cells as pro-lesion cells that promote the growth of lesions once endometrial cells have attached to the peritoneal tissue [16].

#### 2.1.2. Natural killer cells

NK cells exist in one of two states: resting NK cells, denoted by $K_{0}$, and activated NK cells, denoted by $K_{A}$. We model both NK dim and bright cells simply as activated NK cells with moderate cytokine production and cytotoxicity.

*Resting NK cells*: Resting NK cell turnover is through a balance of steady recruitment from the bloodstream and loss through natural cell turnover and activation. We assume $K_{0}$ cells have insignificant cytokine production and cytotixicity compared with their activated form, $K_{A}$.

*Activation of NK cells*: NK cell activation occurs through pro-inflammatory cytokines, such as interleukin IL-12, and interferons IFN-α and IFN-γ [14,49,50]. We assume the main producers of these are macrophages, particularly IL-12 which is produced at high levels by M1-type macrophages [27]. Consequently we assume that $M_{1}$ cells upregulate activation of $K_{A}$ cells.

*Activated NK cell action*: Activated NK cells are assumed to be responsible for cytokine release, cell clearance and NK proliferation. This is modelled as upregulation of M1-type polarization (through IFN-α [14,22]), clearance of endometrial cells and proliferation of activated NK cells (through IL‐2 [49]).

*Activated NK cell clearance*: Activated NK cells have a natural turnover rate similar to resting NK cells. Additionally, activated NK cells are known to become exhausted from the execution of cell clearance [51], after which they no longer contribute to cell clearance. To accommodate this, we model $K_{A}$ to have a higher cell loss rate than $K_{0}$ upon exposure to endometrial cells.

#### 2.1.3. Endometrial cells

We define the endometrial cells to be in three states: eutopic (within the uterus, $E_{0}$), in (peritoneal) fluid ($E_{F}$), and attached (to the peritoneum and/or external surface of the reproductive system/gut, $E_{A}$). $E_{A}$ cells are early lesion cells which then progress to endometriosis. Transitions between the three cell states are strictly sequential (i.e. an attached endometrial cell will never detach).

*Eutopic endometrial cells*: These are endometrial stromal and epithelial cells located on the internal surface of the uterine cavity. The functionalis layer of the endometrium sheds during menstruation, with stromal and epithelial cells found in menstrual fluid. These cells are cleared from the uterine cavity through ‘normal’ menstruation (cleared through cervix) or enter the peritoneal cavity through retrograde menstruation, where they become fluid cells, $E_{F}$.

*Fluid and attached cell turnover*: When cells enter the peritoneal cavity through retrograde menstruation, they are first collected within the peritoneal fluid, and hence we denote them to be in the ‘fluid’ cell state. The fluid cells attach to the peritoneum, from which they can form a lesion. Both fluid and attached cells are cleared through natural cell turnover and clearance initiated by the immune system. We assume the same immune clearance rate for fluid and attached cells. Attached cells proliferate to form lesions, and this proliferation is upregulated by the presence of $M_{2}$ cells.

*Endometrial cell action*: Fluid and attached endometrial cells are detected by macrophages, and their presence results in activation of resting macrophages to an $M_{1}$ state.

### 2.2. Model equations

Using the assumptions described above, we construct a system of ordinary differential equations (ODEs). Upregulation terms follow either mass-action or Hill kinetics, and proliferation and production terms are modelled as logistic growth. The cyclic function for eutopic ($E_{0}$) endometrial cell influx, $\mu_{E}(t)$ (equation (2.9)), was fitted to normalized menstrual cycle volume data [52]. Below, we present the system of equations, and describe the terms that govern cell dynamics and interactions. The cell dynamics under the default parameter values are shown in figure 1b. Parameter values are given in electronic supplementary material, S1.

#### 2.2.1. Macrophage cell dynamics

Equations (2.1) to (2.3) describe resting macrophage ($M_{0}$), *M*1-type macrophages ($M_{1}$) and *M*2-type macrophages ($M_{2}$) dynamics, respectively.


(2.1)dM0dt=μM⏟Circulating M0+ηM[1−(M0+M1+M2MC)][M0+M1+M2]⏟M0 production−θM(KACKA+KA)M0⏟M1 activation, upregulated via KA−β1(EF+EA)M0⏟M1 activation via EF and EA detection−β2M0⏟M2 activation−δM0M0⏟M0 turnover,(2.2)dM1dt=β1(EF+EA)M0⏟M1 activation via EF and EA detection+θM(KACKA+KA)M0⏟M1 activation, upregulated via KA+β21M2⏟M2 repolarization to M1−β12M1⏟M1 repolarization to M2−δM1M1⏟M1 turnover,(2.3)dM2dt=β2M0⏟M2 activation+β12M1⏟M1 repolarization to M2−β21M2⏟M2 repolarization to M1−δM2M2⏟M2 turnover.

Here, $\mu_{M}$ is the supply rate of $M_{0}$ from circulation; $\eta_{M}\left[1-\left(\frac{M_{0}+M_{1}+M_{2}}{M_{\text{C}}}\right)\right]\left[M_{0}+M_{1}+M_{2}\right]$ describes $M_{0}$ production, with carrying capacity $M_{C}$ and rate $\eta_{M}$; $\theta_{M}\left(\frac{K_{A}}{C_{K_{A}}+K_{A}}\right)M_{0}$ describes $M_{0}$ activation to $M_{1}$, upregulated by $K_{A}$, with rate $\theta_{M}$; $\beta_{1}\left(E_{F}+E_{A}\right)M_{0}$ describes $M_{0}$ activation to $M_{1}$, upon detection of $E_{F}$ and $E_{A}$, with rate $\beta_{1}$; $\beta_{2}M_{0}$ describes $M_{0}$ activation to $M_{2}$ with rate $\beta_{2}$; $\beta_{21}M_{2}$ describes repolarization of $M_{2}$ to $M_{1}$, and $\beta_{12}M_{1}$ repolarization of $M_{2}$ to $M_{1}$; $\delta_{M_{0}}M_{0}$ describes the natural turnover of $M_{0}$, and similarly for $\delta_{M_{1}}M_{1}$ and $\delta_{M_{2}}M_{2}$.

#### 2.2.2. Natural Killer cell dynamics

Equations (2.4) and (2.5) describe resting ($K_{0}$), and activated ($K_{A}$) NK cell dynamics, respectively.


(2.4)dK0dt=μK⏟Circulating K0−θKK0(M1CM1+M1)⏟KA activation via M1−δK0K0⏟K0 turnover,(2.5)dKAdt=θKK0(M1CM1+M1)⏟KA activation via M1+ηK(1−KA+K0KC)KA⏟KA proliferation−σ(EF+EA)KA⏟KA exhaustion, via EF, EA interaction−δKAKA⏟KA turnover.

Here, $\mu_{K}K_{0}$ is the supply rate of $K_{0}$ from circulation; $\theta_{K}K_{0}\left(\frac{M_{1}}{C_{M_{1}}+M_{1}}\right)$ describes $K_{0}$ activation to $K_{A}$, upregulated by $M_{1}$, with rate $\theta_{K}$; $\eta_{K}\left(1-\frac{K_{A}+K_{0}}{K_{C}}\right)K_{A}$ describes $K_{A}$ proliferation, with carrying capacity $K_{C}$ and rate $\eta_{K}$; $\sigma \left(E_{F}+E_{A}\right)K_{A}$ describes $K_{A}$ exhaustion, due to $E_{F}$ and $E_{A}$ clearance, with rate σ; $\delta_{K_{0}}K_{0}$ and $\delta_{K_{A}}K_{A}$ describe the natural turnover of $K_{0}$ and $K_{A}$, respectively.

#### 2.2.3. Endometrial cell dynamics

Equations (2.6) to (2.7) describe eutopic ($E_{0}$), in peritoneal fluid ($E_{F}$) and attached ($E_{A}$) endometrial cell dynamics, respectively.


(2.6)dE0dt=μE(t)⏟Endometrial shedding−δE0[ρ0E0⏟Retrograde clearance+(1−ρ0)E0⏟`Normal` clearance],(2.7)dEFdt=δE0ρ0E0⏟Retrograde influx−ω[γKA+(1−γ)M1]EF⏟EF clearance via KA and M1−ρFEF⏟EF attachment−δEFEF⏟EF turnover,(2.8)dEAdt=ρFEF⏟EF attachment+ηEEA(1−EAEC)(M2CM2+M2)⏟EA proliferation upregulated via M2−ω[γKA+(1−γ)M1]EA⏟EA clearance via KA and M1−δEAEA⏟EA turnover,

where


(2.9) $$\begin{array}{r} \mu_{E}(t)=a_{E}{\left[\text{sin}\left(\frac{t+d_{E}}{28}\pi \right)\right]}^{b_{E}}. \end{array}$$

Here, $\mu_{E}(t)$ describes endometrial shedding; $\delta_{E_{0}}\rho_{0}E_{0}$ is $E_{0}$ retrograde clearance to $E_{F}$, and $\delta_{E_{0}}(1-\rho_{0})E_{0}$ is ‘normal’ $E_{0}$ clearance; $\rho_{F}E_{F}$ represents $E_{F}$ attachment, becoming $E_{A}$, with rate $\rho_{F}$; $\omega \left[\gamma K_{A}+\left(1-\gamma \right)M_{1}\right]E_{i}$ describes $E_{F}$ (i=F) and $E_{A}$ (i=A) clearance, through $K_{A}$ and $M_{1}$, with rate ω and γ the proportion of $K_{A}$ relative to $M_{1}$ cell clearance; $\eta_{E}E_{A}\left(1-\frac{E_{A}}{E_{C}}\right)\left(\frac{M_{2}}{C_{M_{2}}+M_{2}}\right)$ describes $E_{A}$ proliferation, with carrying capacity $E_{C}$, upregulated by $M_{2}$, with rate $\eta_{E}$; $\delta_{E_{F}}E_{F}$ and $\delta_{E_{A}}E_{A}$ describe the natural turnover of $E_{F}$ and $E_{A}$, respectively.

#### 2.2.4. Constant influx surrogate model

Taking ${\bar{\mu }}_{E}$ as the average influx over the cycle, we define a surrogate model for the system in which we assume a constant influx into $E_{0}$, i.e.


(2.10) $$\mu_{E}(t)={\bar{\mu }}_{E}.$$

This results in a sufficient match between the constant influx and the cyclic influx systems, in terms of both average quantities and emergent behaviour. The surrogate model provides two advantages to the cyclic influx model: (i) it is computationally more efficient and (ii) it allows for clearer analysis of some aspects of the emergent system dynamics.

#### 2.2.5. Parameters and initial conditions

Unless otherwise specified, the parameters for the model are given in electronic supplementary material, S1, and the response of the system for these parameter values is shown in figure 1b. Where possible, we use parameters from previous within-host immune dynamics models [19,20,53], or estimate them using *in vitro*, *in vivo* or clinical data [5–7,15,17,25,52,54–58]. Due to a lack of quantitative data on immune cell–endometrial cell interactions, several parameters were taken from the cancer and viral literature. Details on the parametrization are given in electronic supplementary material, S2.

To solve the initial value problem for a particular parameter set, we first solve the system of equations using the surrogate model (§2.2.4), whose steady-state solutions inform the initial conditions for the full system with cyclic influx. The initial conditions for the surrogate model solution use a combination of model steady-state values and observations of cell concentrations in the literature [5,25,56] (see electronic supplementary material, S1).

### 2.3. Characterization of disease dynamics

#### 2.3.1. Attachment and disease definitions

We use the value of $E_{A}$ at the end of an endometrial cycle (28 day cycle) to determine if the response results in a diseased state. A low/no disease system is defined as end-of-cycle attachment below some threshold value, ε: $E_{A}(t=28k)<\varepsilon$, $k\in {\mathbb{Z}}_{\geq 0}$, and t>τ, where τ is the initial transient time for the system to reach a cyclic state, for some $\varepsilon \in {\mathbb{R}}_{\geq 0}$.

**Figure 2.**
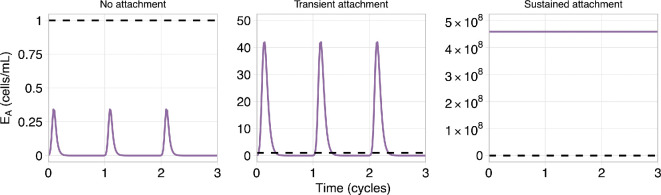
Examples of the three emergent attachment state dynamics observed, assuming a threshold of ϵ=1 cell ml^−1^ (indicated by the black dashed line in the plots). The left two plots (no attachment and transient) are hypothesized to be the low/no disease states, and the right plot (sustained) is hypothesized to be the diseased state.

In addition to classifying responses as high disease or low/no disease, we consider different response states across the endometrial cycle. We classify system dynamics into three attachment states based on the threshold value ε:

(1) low attachment (low/no disease): cell attachment is always below the threshold, $E_{A}(t)\leq \varepsilon \forall t>\tau$;
(2) transient attachment (low/no disease): peak cell attachment is above the threshold but decreases to below the threshold at the end of the cycle, ${\hat{E}}_{A}>\varepsilon ,\;E_{A}(t=28k)\leq \varepsilon \forall k\in \mathbb{Z},t>\tau$;
(3) sustained attachment (diseased): end-of-cycle attachment is above the threshold, $E_{A}(t=28k)>\varepsilon \forall t>\tau$;

where ${\hat{E}}_{A}$ is the maximum $E_{A}$ value over the menstrual cycle, and $E_{A}(t=28k)$ the number of attached cells at the end of the endometrial cycle after initial transient time τ days. The term low/no disease is used as the nature of the modelling paradigm does not permit $E_{A}=0$, for t>0 days. Examples of each of these attachment states are given in figure 2.

#### 2.3.2. Parameters and response metrics of interest

To investigate the questions of interest around endometriosis lesion onset, we examine the model’s response to the variation of three parameters: the proportion of eutopic endometrial cells that are shed retrograde into the peritoneal fluid ($\rho_{0}$), macrophage detection rate of endometrial cells ($\beta_{1}$) and the clearance rate of the endometrial cells by the immune cells (ω). In our model, macrophages’ ability to detect endometrial cells represents the level of apoptotic marker expression and viability of the endometrial cells. M1-type macrophage activation, which occurs predominantly through endometrial cell detection ($\beta_{1}$), and the subsequent activation of NK cells is a pre-requisite to cell clearance (ω). The primary system responses we are interested in are: the number of attached endometrial cells ($E_{A}$) at the end of the cycle, as an indicator for the level of disease; and the level of macrophage activation, measured as the proportion of total macrophages in each activation state $M_{1}$ and $M_{2}$, denoted by ${\tilde{M}}_{1}$ and ${\tilde{M}}_{2}$, respectively, as a comparison with immune profiles observed in patients with endometriosis. We use proportions for macrophage activation to allow for simple comparison with studies on peritoneal fluid cell compositions, which report the proportions of macrophages expressing specific markers.

### 2.4. Code availability

The code to generate the results data is available from GitHub (https://github.com/clairemiller/math_modelling_immune_endo) and archived on Zenodo [59]. The ODE system was simulated using Matlab’s ode15s (R2024b) [60], and the bifurcation analysis was performed using the software AUTO-07p [61].

## 3. Results

### 3.1. Low attachment analysis

We perform an eigenvalue analysis to identify the stability of each of the steady states. By requiring the disease-free steady state to be stable, we find that the following inequality must be satisfied:


(3.1) $$\begin{array}{lcr} & \underset{\text{Upregulation of }E_{A}\text{ proliferation by }M_{2}}{\underbrace{\eta_{E}\frac{M_{2}}{C_{M_{2}}+M_{2}}}}<\underset{E_{A}\text{ clearance via }K_{A}\text{ and }M_{1}}{\underbrace{\omega \left[\gamma K_{A}+\left(1-\gamma \right)M_{1}\right]}}+\underset{E_{A}\text{ turnover}}{\underbrace{\delta_{E}}}, & \end{array}$$

where $\eta_{E}$ is the growth rate of endometrial cells, $C_{M_{2}}$ is the action limiting capacity for $M_{2}$ upregulation of $E_{A}$ proliferation, ω is the lysis rate of $E_{A}$ by the immune cells, with γ representing the proportion of $K_{A}$ relative to $M_{1}$ lysis, and $\delta_{E}$ is the natural clearance rate of $E_{A}$ (see electronic supplementary material, S1, for parameter values). The full analysis is given in electronic supplementary material, S3. The biological interpretation of this disease-free state is that the proliferation rate of the attached endometrial cells, upregulated by $M_{2}$, must be smaller than the clearance rate of endometrial cells—both cell clearance due to the immune response and natural turnover.

This analysis only provides information for when the system is in the disease-free state, and does not provide insight into how a transition to disease may occur.

### 3.2. Hypotheses of disease onset

#### 3.2.1. Retrograde influx

We are interested in understanding whether the observed immune profile of peritoneal fluid from patients with endometriosis could emerge due to the presence of the endometrial cells with no immune dysfunction, and if increased endometrial presence is associated with disease (Hypothesis A). To investigate this, we explore the response of the system as the level of retrograde influx into the peritoneal fluid increases. This is performed by increasing $\rho_{0}$, the proportion of menstrual debris that clears retrograde from the uterus.

**Figure 3.**
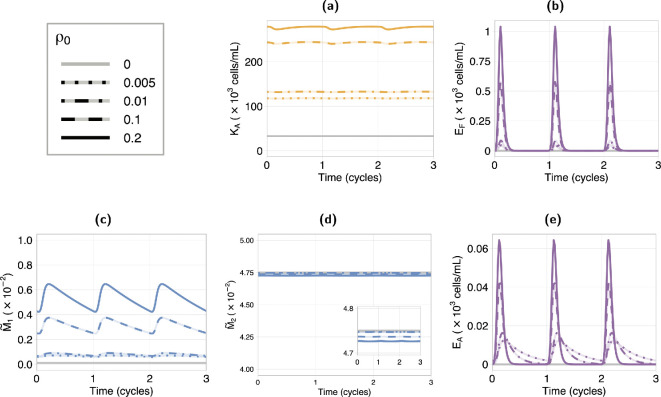
Timeseries of the response for varying endometrial retrograde influx ($\rho_{0}$) shown for three menstrual cycles. We see large peaks in the endometrial cells, both in fluid ($E_{F}$, (b)) and attached ($E_{A}$, (e)) with the menstrual phase of the cycle. Oscillations are also seen in the proportion of macrophage activated to $M_{1}$ type, (c) and NK cell activation, (a). Increased influx causes an increase in the proportion of macrophage activated to $M_{1}$ and activated NK cells, but does not have a significant effect on the proportion of macrophages activated to $M_{2}$, (d). Results show high retrograde influx is associated with a small sustained increase in inflammation but not sustained attachment.

The system response to variations in the retrograde influx is plotted in figure 3. This figure shows the dynamics of the attached endometrial cells and activated immune cells. High levels of retrograde influx are associated with an increase in the proportion of macrophages activated to M1-type (${\tilde{M}}_{1}$, figure 3c) and the concentration of activated NK cells ($K_{A}$, figure 3a), but there is no significant change to the proportion of macrophage activated to M2-type (${\tilde{M}}_{2}$, figure 3d). In our model, $M_{2}$ activation is only indirectly affected by endometrial cell presence, as a result of the subsequent changes in $M_{0}$ and $M_{1}$ levels, which are not large enough here to significantly affect ${\tilde{M}}_{2}$. The increase in ${\tilde{M}}_{1}$, and consequently $K_{A}$, is attributed to the upregulation of $M_{1}$ in response to the presence of $E_{F}$ and $E_{A}$, as described in §2.2.1. We also observe that the relaxation time of $M_{1}$ and $K_{A}$ is longer than the menstrual cycle length, resulting in a sustained state of inflammation. This result aligns with the description of endometriosis as a chronic inflammatory disease [2,22].

We see that a higher retrograde influx results in an increase in the peak level of endometrial cell attachment (figure 3e). Contrary to expectations, low influx leads to higher attachment levels at the end of the cycle (see $\rho_{0}=0.005$, dotted line in figure 3e). This is because an increased influx of endometrial cells causes an increased activation of $M_{1}$ cells, and consequently $K_{A}$ cells, as noted above. As a result, immune clearance of the endometrial cells is increased. At low levels of $\rho_{0}$, there is a decrease in the activation of $M_{1}$ cells, and consequently a decrease in the immune clearance of endometrial cells. The proliferation rate of the attached endometrial cells is comparable between the different influx levels, as increasing $\rho_{0}$ has an insignificant effect on the $M_{2}$ cell levels. Consequently, the low influx systems maintain a low level of sustained attachment while high influx systems exhibit only transient attachment.

Therefore, we see that our results show an increase in retrograde influx is associated with a small, sustained increase in inflammation, but is not associated with increased disease, opposing Hypothesis A.

#### 3.2.2. Immune dysfunction

There are several hypotheses regarding immune system dysfunctions in endometriosis pathogenesis. Two immune dysfunctions we explore are altered detection (Hypothesis B1, model parameter $\beta_{1}$) and clearance (Hypothesis B2, model parameter ω) of the endometrial cells. We consider a reduction in clearance rate by both activated immune cell types, $M_{1}$ and $K_{A}$, noting that NK cells are assumed to be the main clearance cell in the model. Detection dysfunction in our model only applies to macrophages as we assume they are the predominant cell type responsible for detecting the endometrial cells. Importantly, detection dysfunction also has a subsequent effect on cell clearance, through a reduction in immune cell pro-inflammatory activation, which is a pre-requisite for cell clearance.

**Figure 4.**
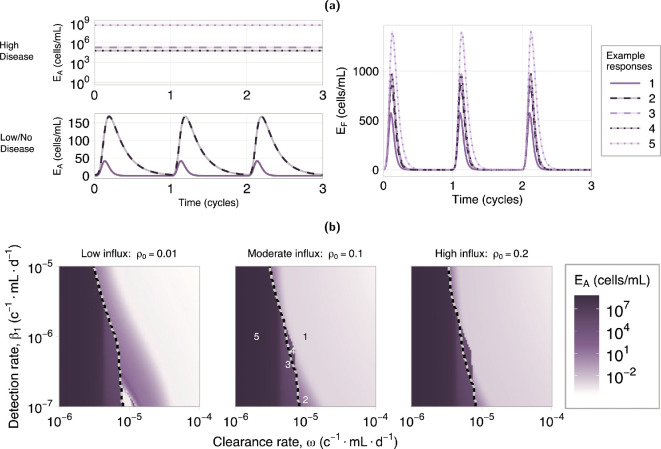
Endometrial attachment and in fluid response to immune dysfunction. (a) Example responses of attached and fluid endometrial cell dynamics at locations indicated on the heatmaps in (b) (moderate influx). (a) Top left: the $E_{A}$ response for the high attachment (high disease) examples. (a) Bottom left: the $E_{A}$ response for the low attachment (low/no disease) examples. (a) Right: the level of endometrial cells in fluid, $E_{F}$, which we observe are always cleared by the end of each cycle. (b) Heatmap showing the level of attached endometrial cells at the end of a cycle. The dashed line indicates the partition between high and low attachment regions according to the inequality in equation (3.1). Units (${\text{c}}^{-1}\text{mL }{\text{d}}^{-1}$) are short for (${\text{cells}}^{-1}\text{mL }{\text{day}}^{-1}$). These results indicate that reduced immune clearance ability is a stronger driver of disease compared with reduced detection ability.

Five example responses for moderate retrograde influx are shown in figure 4a. Examples 1 and 5 in figure 4a show a healthy and diseased response, respectively. In the healthy response both $E_{A}$ and $E_{F}$ are cleared by the end of the cycle, while in the diseased response, only $E_{F}$ is cleared, and $E_{A}$ has a high level of sustained attachment.

The endometrial cell response to variations in both immune detection and clearance ability is shown in figure 4b for three levels of retrograde influx, $\rho_{0}$ (low, moderate and high). A similar investigation was also performed for different levels of endometrial cell attachment rate, which showed attachment rate does not affect these results (see electronic supplementary material, S4). Figure 4b shows the end of cycle (i.e. cycle day 28) values for endometrial attachment ($E_{A}$). We see that, for all $\rho_{0}$ levels, $E_{A}$ transitions from a low attachment state (low/no disease, light purple) to a high attachment state (high disease, dark purple) with decreasing clearance ability. Over a wide range of detection rates, $\beta_{1}$, a reduction in clearance rate, ω, will result in the disease transition. However, for reductions in the detection rate, $\beta_{1}$, this transition only occurs across a narrow range of ω values. This shows the disease transition is dominated by the immune clearance rate, ω.

**Figure 5.**
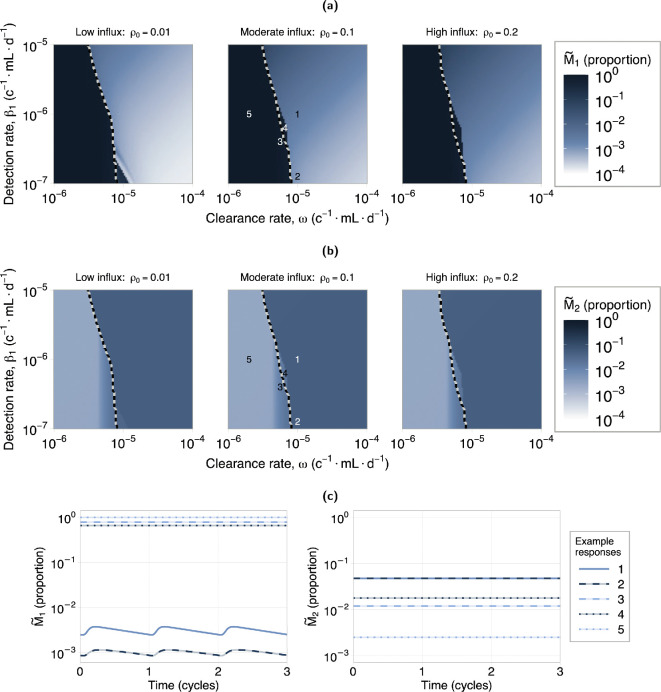
Macrophage activation response to immune dysfunction. (a,b) Heatmaps showing the proportion of macrophage polarized to $M_{1}$ and $M_{2}$, respectively, at the end of a cycle. The dashed line indicates the partition between high and low attachment regions according to the inequality in equation (3.1). Units (${\text{c}}^{-1}\text{mL }{\text{d}}^{-1}$) are short for (${\text{cells}}^{-1}\text{mL }{\text{day}}^{-1}$). (c) Example responses at locations indicated on the heatmaps in (a,b). (c) Left: the proportion of macrophages in an $M_{1}$ activation state. (c) Right: the proportion of macrophages in an $M_{2}$ activation state. Note examples 1 and 2 are overlapping in right plot. The high attachment region is associated with high $M_{1}$ activation and low $M_{2}$ activation. In the low attachment region, decreasing clearance rate is associated with an increase in $M_{1}$ activation while decreasing detection rate is associated with a decrease in $M_{1}$ activation. There is no significant effect on $M_{2}$ activation in the low attachment region.

The dashed lines in figure 4b (and figure 5a,b) partition the regions governed by the inequality in equation (3.1). This partition is calculated using the surrogate model (see §2.2.4). Responses in the right region are predicted to exhibit low attachment behaviour, and those to the left, high attachment behaviour. The alignment between this partition and the disease transition in the numerical results indicates that this relationship is a suitable indicator of disease. The region of disagreement showing high $E_{A}$ but predicted to be in low-disease region is a region where attached endometrial cell proliferation and removal are approximately equal (see example responses 3 and 4 in figure 4a.

Figure 5a,b shows the macrophage activation response to a reduction in immune clearance and detection rate. In both figures, in the high attachment region, there is a high proportion of $M_{1}$ activation and a reduced proportion of $M_{2}$ activation as a result of the high endometrial cell presence. This transition can be seen in the figure 5c, with low ${\tilde{M}}_{1}$ and high ${\tilde{M}}_{2}$ in the two low attachment example responses 1 and 2, and high ${\tilde{M}}_{1}$ and low ${\tilde{M}}_{2}$ for the high attachment example responses 3−5.

In the low attachment region, the results show an increase in ${\tilde{M}}_{1}$ with decreasing clearance ability, while decreasing detection ability results in a decrease in ${\tilde{M}}_{1}$. The latter can be seen in example response 2 in figure 5c, which shows a decrease in ${\tilde{M}}_{1}$ but no change to ${\tilde{M}}_{2}$ compared with example response 1. A decreased detection ability reduces the rate at which macrophages activate into an $M_{1}$ state, consequently leading to this reduction in inflammation.

These results show that a reduced immune clearance rate, ω, leads to high disease and high $M_{1}$ activation. Neither hypothesis alone leads to an increase in $M_{2}$ activation in our results. However, elevated $M_{2}$ activation may occur when $M_{2}$ is upregulated by $E_{A}$, but only once the system already exhibits a high disease state (see electronic supplementary material, S6). Consequently, our results show the reduced clearance hypothesis, Hypothesis B2, is most consistent with the observations of increased macrophage activation in patients with endometriosis compared with both the reduced detection and increased retrograde hypotheses.

### 3.3. Disease transition

We now consider the case, for Hypothesis B, where the immune clearance and detection have a gradual decline in function, rather than a pre-existing dysfunction. This can be explored through a bifurcation analysis in ω and $\beta_{1}$. A bifurcation analysis can be used to determine the equilibrium of a system as model parameters change, and identify points at which the behaviour of these equilibrium changes between being stable, bistable, and unstable, for example. This allows us to identify regions in parameter space that can sustain both a high disease and a low/no disease state (bistability). The observed transition from low to high endometrial attachment in figure 4b with decreased immune function is indicative of bistable system dynamics.

**Figure 6.**
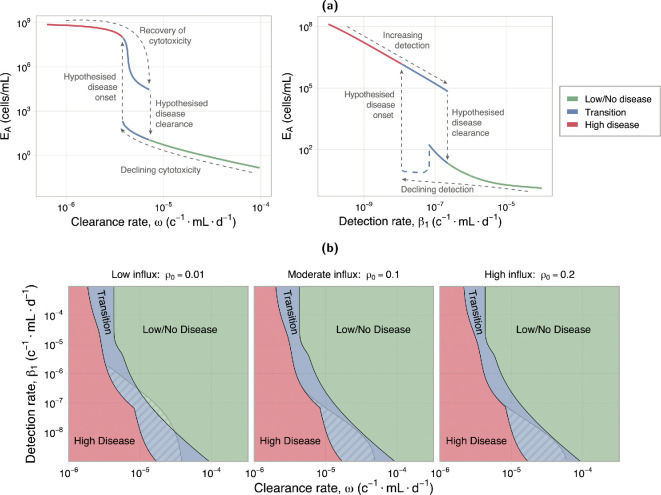
Disease transition plots for clearance and detection rate: (a) clearance (left) and detection (right) rate independently. The dashed blue line (right plot) indicates the solutions reclassified from a limit cycle—showing the median value of $E_{A}^{*}$. (b) Co-dimensional bifurcation in clearance (ω) and detection ($\beta_{1}$) rate. Hatched region shows the reclassified limit cycle solutions. The complete bifurcation diagrams are given in electronic supplementary material, S5. Fixed values for (a) are $\beta_{1}=10^{-6}$${\text{cells}}^{-1}\;\text{ml}\;{\text{day}}^{-1}$, $\omega =10^{-5}$${\text{cells}}^{-1}\;\text{ml}\;{\text{day}}^{-1}$ and $\rho_{0}=0.1$. Units (${\text{c}}^{-1}\;\text{ml}\;{\text{d}}^{-1}$) are short for (${\text{cells}}^{-1}\text{ml}\;{\text{day}}^{-1}$). A patient can start in the disease-free region (green), and then transition through the bistable region (blue), still in low/no disease, but then switch into the high disease state either due to a decline in immune function, or due to additional insult to the system. Disease clearance from this state requires significant improvement to immune function.

We perform a bifurcation analysis, using the surrogate model described in §2.2.4. Figure 6a shows the biologically observable states, from this analysis, of the attached endometrial cells for varying clearance (left) and detection (right) rate. We reclassify solutions containing limit cycles (using the median value of $E_{A}^{*}$, shown as a dashed line in figure 6a, where $E_{A}^{*}$ is a solution containing a limit cycle) as their period of oscillation exceeds the expected timescale of disease onset in the presence of the innate immune response only, beyond which the adaptive immune response is known to play a role [2]. Complete bifurcation diagrams can be found in electronic supplementary material, S5.

For both clearance and detection rate, we observe a bistable regime where the system exhibits hysteresis, which we term the transition region, shown in blue on figure 6a. Biologically, this indicates that a healthy individual can start in a low/no disease state (green); upon an initial decline in immune function, they enter the transition region, remaining in the low/no disease state (bottom blue); with further immune function decline, or other changes to the endometrial or immune cell states, the individual will switch into a high disease state (red, or top blue, respectively), which we hypothesize to be disease onset. The individual is unable to recover from this diseased state with subsequent improvement in immune function (top blue), unless the improvement achieves the hypothesized disease clearance indicated on the figure.

We perform a co-dimensional bifurcation analysis, using the surrogate model, to look at both clearance and detection rate simultaneously. A full co-dimensional bifurcation diagram is provided in electronic supplementary material, S5. The biologically observable states from this analysis are shown in figure 6b for three levels of retrograde influx (low, moderate and high), with solutions containing limit cycles reclassified as described above (hatched region in figure 6b). Regardless of the level of retrograde influx, we observe similar behaviours—changing the influx level simply shifts the transition region in parameter space. This reinforces our finding that increased retrograde influx is neither associated with increased disease, nor a driving factor of system dynamics in our model. Notably, a transition region is always present between the low/no and high disease states. As detailed previously, this indicates that a switch into high disease (disease onset) is not recoverable without significant improvements in immune system function (disease clearance).

## 4. Discussion

In this study, we develop a novel compartmental mathematical model of the innate immune response to endometrial cell influx from retrograde menstruation, to describe the early stages of superficial peritoneal endometriosis lesion onset. We use our model to interrogate two key questions around endometriosis onset. First, could increased endometrial cell presence describe altered immune states observed in patients with endometriosis and lead to disease (Hypothesis A). Second, of two hypothesized disorders: immune system detection and clearance of endometrial cells in menstrual debris (Hypotheses B1 and B2), which is more associated with disease and consistent with clinical observations. This is the first mathematical model that has been developed to interrogate mechanisms of endometriosis lesion onset, highlighting the importance of the immune response.

Our results show that an increase in retrograde menstruation, with no immune system dysfunction, is not associated with increased disease, but is associated with a small, sustained increase in inflammation (figure 3). We find the key driver of disease onset is a decrease in the clearance rate of endometrial cells by immune cells (figure 4), supporting Hypothesis B2. Both decreased clearance rate and high disease are associated with an increase in M1-type macrophage activation, but not M2-type activation (figure 5). This increase is sustained across the cycle and may cause a state of chronic inflammation. A decrease in immune detection rate, which represents reduced phagocytosis and apoptotic marker expression by the endometrial cells, will also result in disease onset within a specific range of clearance rates, indicating that different immune profiles can be indicative of disease. Given an immune profile, we provide a relationship between attached endometrial cell proliferation and removal that is a predictor of disease (equation (3.1)). Through a bifurcation analysis, we show that a transition region exists that dictates the dynamics of disease onset and disease clearance (figure 6). This result describes how, following disease onset, a significant recovery of immune function is required to obtain disease clearance. Our analysis supports a hypothesis that a decline in immune function, in particular immune cell cytotoxicity, such as immune exhaustion resulting from chronic inflammation [62], may be a driver of disease onset. Our results show a sustained increase in inflammation ($M_{1}$), in the low/no disease state, under both the reduced clearance hypothesis and the increased retrograde influx hypothesis. We hypothesize that this sustained M1-type activation would induce a state of chronic inflammation, causing a reduction in immune system efficacy and contributing to disease onset.

A recent review has challenged the hypothesis that frequency and volume of retrograde menstruation is different between patients with endometriosis and controls [8]. This is supported by our results, which show no association with increased retrograde influx and disease (figure 3). Endometrial stem and progenitor cell concentrations in peritoneal fluid have been observed to be one to two orders of magnitude higher during the menstrual phase, compared with non-menstrual phase, in both control and patients with endometriosis [13]. Endometriosis cases had increased variation between patients, with some patients measuring concentrations comparable with control cases, and others measuring significantly higher cell concentrations, which may indicate there is no unique pathway to disease onset. This observation does not support a particular hypothesis based on our results: a significant increase in endometrial cells in fluid can be seen during the menstrual phase for all levels of retrograde influx (figure 3), and our bifurcation analysis results show only small changes in endometrial cells in fluid between low/no disease and high disease, particularly in the transition region (electronic supplementary material, S5). This example demonstrates that, without high precision on cycle time and additional physiological information, single time point data on peritoneal fluid cell concentrations are insufficient to inform mathematical models or indicate the presence of disease.

Several studies have shown increases in both M1- and M2-type macrophages, as proportions of total macrophage, in patients with endometriosis [15–17]. Our results show an increase in M1-type macrophages is associated with increased retrograde menstruation (figure 3c) and decreased immune clearance capabilities (figure 5c); and a decrease in M2-type macrophages is associated with a decrease in immune clearance capabilities (figure 5c). Our model does not predict an increase in both macrophage activation states under any hypothesis. The lack of increased M2-type activation in our results is due to no $M_{2}$ upregulation mechanism in the model. By incorporating $M_{2}$ upregulation by $E_{A}$, a high disease state, characterized by both increased $M_{1}$ and $M_{2}$ activation, may occur (see electronic supplementary material, S6). However, this upregulation alone is not sufficient to trigger disease onset. It is important to also note that clinical data is only collected after confirmed diagnosis, which is usually several years after disease onset [1,63]. This leads to ambiguity in which observations of increased M1- or M2-type activations are associated with the early stages of disease and those that emerge during later stages of lesion development and maintenance.

Few studies have focused on NK cell concentrations and activation types in peritoneal fluid. A significant increase (approximately fourfold) in the proportion of leukocytes that stained for NHK-1 in stage I disease has been observed [6], which could indicate an increase in the concentration of activated NK cells. Such an increase is predicted by our model under the increased retrograde menstruation hypothesis, and the decreased cell clearance hypothesis in the low disease state (see electronic supplementary material, S5).

To our knowledge, this is the first mechanistic mathematical model developed in the context of endometriosis. Our model combines understanding of macrophage, NK cell and endometrial cell dynamics. Our model provides a framework that can be used to provide insight into open questions, such as those considered in this article, around system changes that contribute to disease onset compared with those that emerge due to the disease. It can also be used to better understand the interacting role of different mechanisms hypothesized through *in vitro* models within an *in vivo* environment.

There are several known modelling limitations to this work. We use a deterministic model to describe interactions and transitions, meaning that stochastic effects, such as menstrual cycle variation, low cell numbers and delays in immune onset, are not accounted for. The cell transitions and interactions are based on understanding of immune interactions from the endometriosis, cancer and viral literature, as there is limited understanding of interactions between immune cells and endometrial cells. As with many models, there is also little quantitative knowledge of the system, particularly for the early stage of lesion onset. This is in part due to the extensive delays patients experience before they receive a clinical diagnosis for endometriosis [63]. Our model has highlighted the need for several quantitative data requirements. In particular, longitudinal studies of patients that capture time-series immune profiles, and connected datasets that provide full peritoneal fluid immune cell profiles of individuals, and also record menstrual cycle day at time of collection. In the future, we will explore opportunities around parameter estimation using clinical and experimental data. For example, our model would greatly benefit from *in vitro* studies of activation states and clearance rates using co-culture assays, animal models or organoids to measure apoptosis or immune activation markers over time. Future work could also leverage single-cell gene profiling studies [64,65], alongside an extension of our model that focuses on specific macrophage cell subtypes, to perform parameter estimation. This would require the development of a comprehensive method for linking the identified cell clusters in the data to model cell activation states or associated cell activities.

We have focused our investigation on the innate immune system (though it is known that the adaptive system also plays a role [2]), and dysfunctions related to early-stage disease and the pro-inflammatory immune response. We assume a binary classification of macrophage activation is sufficient to capture the average behaviour of the macrophage activation spectrum: macrophage activation and phenotype dynamics would merit its own modelling investigation before inclusion in this model. Additionally, we did not distinguish between the different endometrial cell types, such as stromal fibroblasts, which have been implicated in disease. Activated fibroblasts have been associated with a pro-inflammatory phenotype and show spatial correlation with macrophage populations in the lesion microenvironment, as well as progesterone resistance [36,37]. Inclusion of the role of these cells in the model could have the effect of increasing the inflammation and macrophage profiles observed in our results, consequently increasing the inflammatory response observed, particularly under the increased retrograde hypothesis. We expect this is most important during the lesion development stage. Further innate immune cells that may also play a role include neutrophils, mast cells and dendritic cells [2]. Future work will extend the model to include (i) hormonal effects on immune and endometrial cell behaviours, (ii) influx of immune cells into the peritoneal fluid as a component of the menstrual debris, (iii) investigations into interactions between specific endometrial cell types and the immune cells, and (iv) explicitly modelling the production and action of particular pro- and anti-inflammatory factors (including cytokines). This will allow us to investigate hypotheses of disease onset relating to progesterone resistance of the endometrial cells [46,47], hormonal effects on the immune cells [22], abnormal immune function in eutopic endometrium [35,66] and the effect of altered cytokine production or response on disease onset [3,14,21,31]. With further development and validation, a model such as this could be used for personalized medicine applications. By collecting individual-specific measurements for key parameters it will be possible to develop a risk profile for their susceptibility to disease, or determine the efficacy of potential treatments for preventing lesion onset and growth.

There are many unanswered questions regarding disease onset in endometriosis pathophysiology, including contradicting evidence between different studies and uncertainties about mechanisms that drive disease onset versus those that result from the disease. Our study uses mathematical modelling to provide insight into the role of the innate immune system in superficial peritoneal endometriosis lesion onset. Results show that increased retrograde influx results in small changes to immune profiles but is not associated with disease. Immune dysfunction does lead to disease onset according to our model, in particular a dysfunction in immune clearance ability, and recovery from disease requires significant improvements to immune function. Our model is ideally placed to provide better understanding towards the role of different mechanisms hypothesized through *in vitro* models within an *in vivo* context. With further development and robust parametrization methods, mathematical modelling will facilitate the identification of potential biomarkers and immunotherapies for endometriosis, and improve our understanding of between-patient variation, moving towards the goal of personalized medicine.

## Data Availability

The code to reproduce the results is archived on Zenodo [59]. Electronic supplementary material is available online [67].
